# The LILI Motif of M3-S2 Linkers Is a Component of the NMDA Receptor Channel Gate

**DOI:** 10.3389/fnmol.2018.00113

**Published:** 2018-04-06

**Authors:** Marek Ladislav, Jiri Cerny, Jan Krusek, Martin Horak, Ales Balik, Ladislav Vyklicky

**Affiliations:** ^1^Department of Cellular Neurophysiology, Institute of Physiology of the Czech Academy of Sciences, Prague, Czechia; ^2^Department of Physiology, Faculty of Science, Charles University in Prague, Albertov, Czechia

**Keywords:** glutamate receptor gating, electrophysiology, spontaneous activity, channel open probability, protein block alphabet, molecular modeling

## Abstract

N-methyl-D-aspartate receptors (NMDARs) mediate excitatory synaptic transmission in the central nervous system, underlie the induction of synaptic plasticity, and their malfunction is associated with human diseases. Native NMDARs are tetramers composed of two obligatory GluN1 subunits and various combinations of GluN2A-D or, more rarely, GluN3A-B subunits. Each subunit consists of an amino-terminal, ligand-binding, transmembrane and carboxyl-terminal domain. The ligand-binding and transmembrane domains are interconnected via polypeptide chains (linkers). Upon glutamate and glycine binding, these receptors undergo a series of conformational changes leading to the opening of the Ca^2+^-permeable ion channel. Here we report that different deletions and mutations of amino acids in the M3-S2 linkers of the GluN1 and GluN2B subunits lead to constitutively open channels. Irrespective of whether alterations were introduced in the GluN1 or the GluN2B subunit, application of glutamate or glycine promoted receptor channel activity; however, responses induced by the GluN1 agonist glycine were larger, on average, than those induced by glutamate. We observed the most prominent effect when residues GluN1(L657) and GluN2B(I655) were deleted or altered to glycine. In parallel, molecular modeling revealed that two interacting pairs of residues, the LILI motif (GluN1(L657) and GluN2B(I655)), form a functional unit with the TTTT ring (GluN1(T648) and GluN2B(T647)), described earlier to control NMDAR channel gating. These results provide new insight into the structural organization and functional interplay of the LILI and the TTTT ring during the course of NMDAR channel opening and closing.

## Introduction

Excitatory signal transduction in the mammalian brain is primarily mediated by glutamate-activated ionotropic receptors (iGluRs) represented by α-amino-3-hydroxyl-5-methyl-4-isoxazole-propionate (AMPA), kainate and N-methyl-D-aspartate receptor (NMDAR) subtypes (Dingledine et al., 1999; Traynelis et al., 2010). Virtually all CNS circuits employ NMDAR-mediated excitatory postsynaptic current to regulate physiological functions and, in addition, this component has been implicated in various forms of synaptic plasticity thought to underly learning and memory formation (Dingledine et al., 1999; Lynch, 2004). In addition to NMDAR-mediated postsynaptic Ca^2+^ current, unconventional presynaptic NMDARs were identified at several types of synapses, where they modulate presynaptic neurotransmitter release (Dore et al., 2017; Bouvier et al., 2018). Part of this NMDAR action is Ca^2+^-insensitive metabotropic signal transduction where a conformation change in the cytoplasmatic part of receptor activates an intracellular signaling pathway. Postsynaptically, metabotropic NMDAR signaling is capable to trigger long-term depression of synaptic transmission (Nabavi et al., 2013; Dore et al., 2016).

Native NMDARs are tetramers assembled from two obligatory glycine-binding GluN1 subunits in combination with two glutamate-binding GluN2A-D or glycine-binding GluN3A-B subunits (Ulbrich and Isacoff, 2008; Traynelis et al., 2010). Like other iGluR subunits, NMDAR subunits exhibit a conserved domain organization. The most distal part to the cell membrane is the amino-terminal domain linked to the ligand-binding domain (LBD), which is connected to the transmembrane domain (TMD) which, in turn, is connected to the intracellular carboxy domain (Traynelis et al., 2010). The clamshell-like LBD (S1 and S2 lobes) and the TMD, composed of M1-M4 transmembrane helices, are central to receptor function. The heteromeric LBD dimer forms a tight interface at the level of the S1 lobes while the bottom S2 lobes remain relatively mobile without a significant intradimer dimerization contact. Interdimer interfaces encompass residue contacts in upper (S1) and lower (S2) lobe (Furukawa et al., 2005; Karakas and Furukawa, 2014; Lee et al., 2014). Agonist binding evokes movement at the level of the S2 lobe (Furukawa et al., 2005; Yao et al., 2013) and it was suggested that agonist-induced conformation changes impact three polypeptide linkers connecting to the TMD forming the ion channel (Karakas and Furukawa, 2014). Recent structural data indicate that NMDAR agonist activation involves a rearrangement of inter-LBD interfaces (Tajima et al., 2016; Zhu et al., 2016). Based on the analysis of the available crystal structures of NMDAR and of isolated LBD structures, the clamshell-like displacement of the S2 with respect to the S1 is relatively small and insufficient to explain the magnitude of the displacement at the level of the TMD.

Channel opening is the key step in the NMDAR gating that allows the flux of ions including Ca^2+^ across the membrane. Crystal structures of the NMDAR with glutamate and glycine bound indicate that the M3 helices of the TMD tightly surround the central ion channel axis (closed conformation) and the M1 and M4 helices are much less packed at the periphery (Karakas and Furukawa, 2014; Lee et al., 2014). Several lines of evidence indicate that a rearrangement of M3 helices in the activated conformation of the receptor makes the central cavity of the channel accessible to ions, therefore implying a crucial role of the M3-S2 linkers in channel opening (Sobolevsky et al., 2004).

Modular composition, with linkers connecting functional domains, is a common characteristic of membrane-located ion channels. For BK potassium channel it has been shown that the length of the linker between the ion channel and the intracellular regulatory domain impacts channel activity. The probability of channel opening increased in presence of a shorter linker and decreased when the linker was extended by additional amino acids (AA; Niu et al., 2004). The concept of mechanical coupling between the LBD and the M3 helix in ionotropic glutamate receptors has been tested by artificial extension of GluN1 and GluN2A M3-S2 linkers with glycine residues inserted into the distal part of the linkers (in the proximity of the LBD) and the activity of mutated NMDAR was significantly impaired (Kazi et al., 2014). Open probability (Po) of the ion channel decreased with the extended linker length; however, this was subunit specifical, indicating distinct roles of the two subunits in the process of channel opening, where the GluN1 subunit mainly regulates the duration of the opening while the GluN2 subunit impacts the frequency of opening (Kazi et al., 2014). The opposite approach, where the length of the linker was reduced by deletion (GluN1(ΔT648) and GluN2A(ΔT644)) the Po was unaffected or decreased, respectively. Simultaneously performed homology modeling led to ambiguous results (Kazi et al., 2014). Moreover, previously published data showed that mutations of specific amino acids in the M3 helix and its proximity (the TMD part of the M3-S2 linker) induce various degrees of spontaneous NMDAR activity. Constitutively open channels were identified in SYTANLAAF motif (*S* = 1, *F* = 9) where T3A, A4C and A7C were mutated in the GluN1 subunit, and A3C and A7C in GluN2 subunits (Jones et al., 2002; Kashiwagi et al., 2002; Sobolevsky et al., 2007; Chang and Kuo, 2008; Xu et al., 2012).

The structure of the M3-S2 linkers and the related ends of the M3 helices has not been determined with high resolution in the solved crystal and cryo-EM structures, preventing clear understanding of how their structure and function contribute to the ion channel gating at the atomic level (Karakas and Furukawa, 2014; Lee et al., 2014; Tajima et al., 2016; Zhu et al., 2016). Structural data, however, indicate that the M3-S2 linkers adopt different orientations—while the GluN1 M3-S2 linkers are almost parallel with the longitudinal receptor axis, the GluN2 M3-S2 linkers are parallel with the membrane (Karakas and Furukawa, 2014; Lee et al., 2014). This suggests different roles of the GluN1 and the GluN2 subunits in channel gating and may contribute to the understanding of the specific molecular mechanism of allosteric coupling between the glutamate- and glycine-binding sites (Mayer et al., 1989).

The goal of this study was to determine the role of the M3-S2 linkers in NMDAR gating. Our results indicate that a novel structural motif formed by two pairs of GluN1(L657) and GluN2B(I655) residues (LILI motif) located at the transition of M3 helices to S2 linkers are functionally linked with the TTTT ring located within the NMDAR channel pore to control channel gating.

## Materials and Methods

### Transfection and Maintenance of Cells

Human embryonic kidney (HEK 293T) cells (American Type Culture Collection, ATTC No. CRL1573, Rockville, MD, USA) were cultured in Opti-MEM I (Invitrogen, Carlsbad, CA, USA) with 5% fetal bovine serum (PAN Biotech, Aidenbach, Germany) at 37°C in 5% CO_2_. The day before transfection, the cells were plated in 24-well plates at a density of 2 × 10^5^ cells per well. The next day, the cells were transfected with expression vectors containing wild-type (WT) or mutated glutamate receptor subunit GluN1–1a (GluN1; GenBank accession no. U08261) and GluN2B(GenBank accession no. M91562) and GFP (green fluorescent protein; pQBI 25, Takara, Tokyo, Japan) genes. Briefly, equal amounts (0.25 mg) of cDNAs encoding for GluN1, GluN2B and GFP were mixed with 0.9 μL of Matra-A Reagent (IBA, Göttingen, Germany) and added to confluent HEK 293T cells. After trypsinization, the cells were resuspended in Opti-MEM I containing 1% fetal bovine serum supplemented with 15 mM MgCl_2_, 1 mM D, L-2-amino-5-phosphonovaleric acid and 1 μM ketamine, and plated on 30 mm poly-L-lysine-coated glass coverslips. Transfected cells were revealed by GFP epifluorescence.

Site-directed mutagenesis was performed using the QuikChange Site-Directed Mutagenesis Kit (Agilent Technologies, Santa Clara, CA, USA) in accordance with the instructions of the manufacturer, using manually designed primers purchased from Sigma-Aldrich. DpnI-treated PCR reaction was transformed into competent XL10-Gold *E. coli* cells, positive clones were selected, and isolated DNA plasmids were sequenced. All mutations were verified by DNA sequencing (SEQme, Dobris, Czech Republic and/or Eurofins Genomics, Germany). Amino acids are numbered according to the full-length protein, including the signal peptide, with the initiating methionine as number 1.

### Electrophysiological Recording

Experiments were performed 18–48 h after the end of transfection on cultured HEK 293T cells transfected with vectors containing GluN1/GluN2B/GFP. Whole-cell and single-channel activity were recorded at room temperature using a patch-clamp amplifier (Axopatch 200A; Molecular Devices, Sunnyvale, CA, USA). Agonist-induced whole-cell responses were low-pass filtered at 2 kHz with a 4-pole Bessel filter (Frequency Devices, Haverhill, MA, USA), sampled at 5 kHz, and analyzed using pClamp software version 10 (Molecular Devices). Patch pipettes (3–6 MΩ) were pulled from borosilicate glass and filled with an intracellular solution containing (in mM): 120 gluconic acid, 15 CsCl, 10 BAPTA, 10 HEPES, 3 MgCl_2_, 1 CaCl_2_ and 2 ATP-Mg^2+^ salt (pH adjusted to 7.2 with CsOH). Extracellular solution (ECS) contained the following (in mM): 160 NaCl, 2.5 KCl, 10 HEPES, 10 glucose, 0.2 EDTA and 0.7 CaCl_2_ (pH adjusted to 7.3 with NaOH).

Steady-state single-channel recordings using the cell-attached patch-clamp technique at room temperature were analog-filtered at 10 kHz with a 4-pole Bessel filter and sampled at 25 kHz. Standard bath solution was the same as the control solution in the whole-cell recording. Thick-wall borosilicate patch pipettes were pulled and fire-polished achieving resistances between 12 MΩ and 25 MΩ when measured in the bath and filled with an ECS containing (in mM): 160 NaCl, 2.5 KCl, 1 mM EDTA and 10 HEPES (pH-adjusted to 8.0 with NaOH) as well as 0.1 glycine and 1 glutamate. Recordings were performed at a very low concentration of protons and in the absence of divalent ions (1 mM EDTA) to negate any differences in sensitivity to these modulators (Popescu and Auerbach, 2003; Amico-Ruvio and Popescu, 2010). Inward openings were detected by applying a pipette potential of +100 mV.

Analysis of single-channel cell-attached records was performed in Nicolai and Sachs (2013). According to Colquhoun and Hawkes (1990), we chose records that were sufficiently long (>5000 events) and entirely free of overlapping openings, which indicated high confidence (99%) of measuring single-channel activity. The lowest Po we observed was 0.03. For this Po, simultaneous opening of two channels should be visible approx. every 140 events, which is over 35-fold fewer events than our chosen minimum. The data were idealized with an SKM algorithm, digitally low-pass filtering at 12.5 kHz and applying 0.12 ms (3 samples) dead time. Analysis of idealized data was performed with the maximum interval likelihood (MIL) algorithm (Qin et al., 1997; Amico-Ruvio and Popescu, 2010). Po, mean open time (MOT) and mean closed time (MCT) values were averaged for each construct and compared with each other.

### Homology Modeling

We have used the recently available crystal structures (PDB IDs—4tll, 4tlm and 4pe5; Karakas and Furukawa, 2014; Lee et al., 2014) as templates for homology modeling of an all-atom complete structure including the linker region using the MODELLER 9v14 suite of programs (Sali and Blundell, 1993). The crystal structures carry several sequence modifications, including deletions, substitutions and introduction of disulfide bridges in order to stabilize the interaction between amino-terminal domains as well as in the TMD region. The overall NMDAR crystal structure quite likely reflects liganded receptor with the channel in the closed state. The homologous sequences were aligned using MUSCLE and visually inspected within Unipro UGENE program. The residues missing in the template structures were modeled using the “automodel” function of MODELLER, including symmetry restraints for the Cα atoms of corresponding pairs of subunits.

Studied mutations (substitutions and deletions) within the linker region were introduced using the MODELLER package, based on the WT homology model. The complete atomic models of GluN1/GluN2B receptor and its mutants were further used for large-scale molecular modeling of linkers.

### Conformational Sampling

Conformational sampling and refinement of linker regions was performed using the “loopmodel” function of MODELLER without any symmetry restraints, allowing a different structure for each linker. The full conformational space of selected linkers is too complex to be efficiently sampled by a classical molecular dynamics simulation. We have thus employed a Monte Carlo-like procedure, randomizing and refining the linker structure based on the simplified atomistic force field of MODELLER, which is capable of reproducing the crystal orientation and native contacts of a randomized loop for known structures. The “loopmodel” function involving the “refine.fast” routine was used for this enhanced sampling. The whole linker sequence LVLDRPEERITGIND of GluN1 and MIQEEYVDQVSGLSD sequence of GluN2B was selected for the refinement. Further, the shorter patches LVLDRPEE and MIQEEYVD, close to the TMD, were also modeled separately. For each WT and mutated linker region, we have performed 1000 “loopmodel” runs with different random seed setting and the Discrete Optimized Protein Energy (DOPE) score for each model was calculated. The DOPE score is based on a statistical potential optimized for model assessment. The DOPE score is designed to select the best structures from a set of MODELLER prepared models. For the NMDAR structure containing two GluN1 and two GluN2B subunits, this produced 2000 models of each region.

### Analysis of Conformations

The complex ensemble of structures for each linker variant was further analyzed in terms of Protein Blocks (PB; de Brevern et al., 2000) assigned using the PBxplore package (Barnoud et al., 2017). For easier PB “sequence” processing, three “letters” of the alphabet were redefined to contain only standard one letter AA codes (B → Q, J → S, O → R) allowing us to use common sequence analysis tools. For each model, we have assigned its conformational descriptors in terms of the PB structural alphabet. PB represents a more detailed extension of the commonly used helix/sheet/coil secondary structure classification while still allowing conversion of complex 3D structural data into a simple 1D sequence-like data. The 2000 PB “sequences” for each sampled linker region sequence were combined and their structural content plotted using the weblogo program version 3.3 employing a user-defined color palette. The results from a separate run producing 1000 models were qualitatively identical to the larger run, proving the extent of simulations as sufficient.

### Visualization of Structures

Graphical representation and analysis of residues surrounding selected regions were performed using PyMOL version 1.8.3 and VMD version 1.8.7.

### Statistical Analysis

All data are expressed as mean ± SEM. Statistical differences between two groups of data was evaluated using a *t*-test and *P* < 0.050 was taken as the limit of significance. Multiple comparisons were made using one-way analysis of variance (ANOVA) and a Dunnett’s *post hoc* analysis was applied only when a significant (*P* < 0.050) effect was evident (SigmaStat v3.5).

## Results

### Deletions in the M3-S2 Linkers Promote Ion Channel Opening

**Figure 1:**
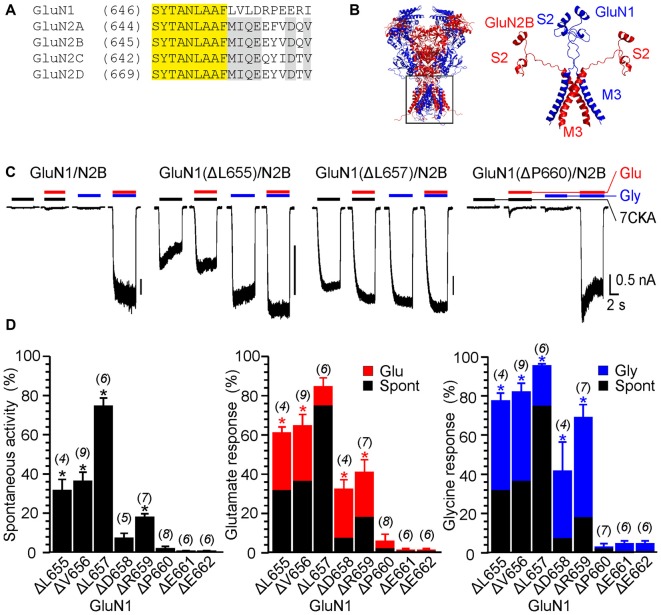
The effect of single-deletion mutations of GluN1 linker on receptor activity. **(A)** Amino acids (AA) sequence alignment for the outer segment of the pore-forming region of the M3 and the initial segment of the M3-S2 linker. AA number is given in parentheses. Conserved motifs in N-methyl-D-aspartate (NMDA) receptor subunits are colored yellow (GluN1/GluN2) and gray (GluN2A-D). **(B)** Homology model of GluN1/GluN2B and the structure of the M3 and M3-S2 linkers are shown. GluN1 is colored in blue and GluN2B is colored in red. Note the different orientation of the M3-S2 linkers in GluN1 and GluN2B. **(C)** Representative current responses of GluN1/GluN2B wild-type (WT), GluN1(ΔL655)/GluN2B, GluN1(ΔL657)/GluN2B and GluN1(ΔP660)/GluN2B. Application of extracellular solution (ECS) containing 7-Chlorokynurenic acid (7CKA; 10 μM) with no added Mg^2+^, glutamate, or glycine is indicated by a black bar (7CKA); application of 1 mM glutamate and 7CKA (10 μM) with no added Mg^2+^ or glycine is indicated by a red bar (Glu); application of 0.1 mM glycine with no added Mg^2+^ or glutamate is indicated by a blue bar (Gly). In between the applications of Mg^2+^-free ECS, the cells were bathed in ECS containing 2 mM Mg^2+^. **(D)** Summary graphs of the mean ± SEM (*n*) of the relative responses—*RI*_Spont_ (black columns), *RI*_Glu_ (red columns) and *RI*_Gly_ (blue columns; see Equations 1–3)—in receptors with a single residue deleted in GluN1. Differences in the relative responses determined for WT and mutated receptors were statistically significant; one-way ANOVA (*P* < 0.001); followed by multiple comparisons of the relative responses of mutated receptors vs. WT (*RI*_Spont_ 0.44 ± 0.06 (*n* = 24); *RI*_Glu_ 0.63 ± 0.30 (*n* = 24); *RI*_Gly_ 1.1 ± 0.2 (*n* = 24)); Dunnett’s test; *P < 0.050.

There is a causal relation between glutamate and glycine binding to the NMDAR and channel opening. Simple mechanistic view suggests that conformational changes in the LBD result in the movement of M3-S2 linkers followed by a centrifugal displacement of GluN1 and GluN2B M3 helices and channel opening. Kazi et al. (2014) have shown that extension in the distal part of the linker (close to the LBD) impaired agonist-dependent channel opening. Therefore, we hypothesized that, in an opposite way, we could control the initial steps of the NMDAR opening and mimic glutamate activation by shortening the GluN2B M3-S2 linkers and glycine activation by shortening the GluN1 M3-S2 linkers. To prove our hypothesis and elucidate the role of the M3-S2 linkers, we made a series of single AA deletions in the GluN1(L655–E662) and GluN2B(M654–D661) M3-S2 linker, in its initial segment located in the proximity of the ion channel vestibule. The relatively sparse structural data for linkers suggest a rather extended polypeptide chain without any loops that would compromise the effect of deletion on the length of linkers (see Figures 1A,B for the homology and orientation). The mutated receptors were screened electrophysiologically.

The experimental protocol consisted of recording responses to a change of the ECS containing 2 mM Mg^2+^ to the solution containing: (i) 10 μM 7-Chlorokynurenic acid (7CKA; a competitive glycine site inhibitor, Kemp et al., 1988) with no added Mg^2+^, glutamate, or glycine (spontaneous activity; *I*_7CKA_); (ii) 1 mM glutamate and 10 μM 7CKA with no added Mg^2+^ or glycine (glutamate-induced responses; *I*_Glu_); (iii) 0.1 mM glycine with no added Mg^2+^ or glutamate (glycine-induced responses; *I*_Gly_); and (iv) 1 mM glutamate and 0.1 mM glycine with no added Mg^2+^ (glutamate and glycine-induced responses; *I*_Gly+Glu_). Spontaneous activity and activity induced by a single agonist is expressed normalized with respect to the responses induced by glutamate together with glycine. Thus, relative spontaneous current (*RI*_Spont_) was defined as: *RI*_Spont_ = *I*_7CKA_/*I*_Glu+Gly_ *(Equation 1)*; relative glutamate responses (*RI*_Glu_) were defined as: *RI*_Glu_ = *I*_Glu_/*I*_Glu+Gly_ – *RI*_Spont_ (*Equation 2*); and relative glycine responses as: *RI*_Gly_ = *I*_Gly_/*I*_Glu+Gly_ − *RI*_Spont_ (*Equation 3*). As expected, in the WT GluN1/GluN2B receptors, spontaneous activity and the responses to only glutamate or only glycine were negligible when compared to those induced by 1 mM glutamate and 0.1 mM glycine (*RI*_Spont_ = 0.44 ± 0.06%; *n* = 24; *RI*_Gly_ = 1.1 ± 0.2%; *n* = 24; *RI*_Glu_ = 0.63 ± 0.30%; *n* = 24; Figure 1C). It is quite likely that this minor activity reflects indirect effect of Mg^2+^ wash out on membrane properties, rather than NMDAR activity.

**Figure 2:**
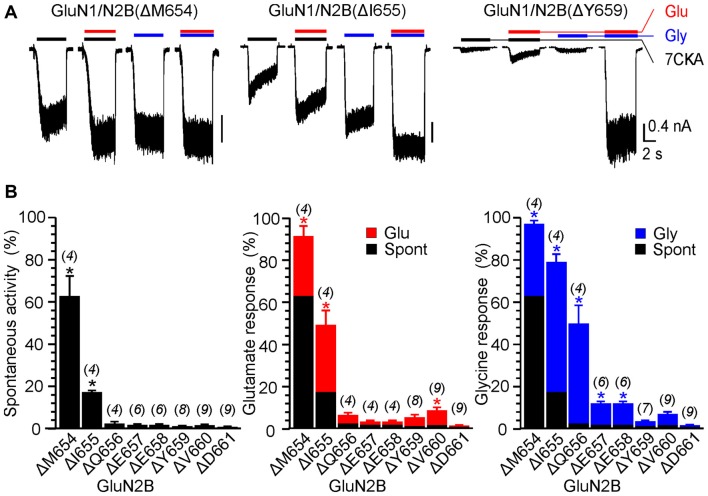
The effect of single-deletion mutations of GluN2B linker on receptor activity. **(A)** Representative current responses of GluN1/GluN2B(ΔM654), GluN1/GluN2B(ΔI655) and GluN1/GluN2B(ΔY659). Application of ECS containing 7CKA (10 μM) with no added Mg^2+^, glutamate, or glycine is indicated by a black bar (7CKA); application of 1 mM glutamate and 7CKA (10 μM) with no added Mg^2+^ or glycine is indicated by a red bar (Glu); application of 0.1 mM glycine with no added Mg^2+^ or glutamate is indicated by a blue bar (Gly). In between the applications of Mg^2+^-free ECS, the cells were bathed in ECS containing 2 mM Mg^2+^. **(B)** Summary graphs of the mean ± SEM (*n*) of the relative responses—*RI*_Spont_ (black columns), *RI*_Glu_ (red columns) and *RI*_Gly_ (blue columns; see Equations 1–3)—in receptors with a single residue deleted in GluN2B. Differences in the relative responses determined for WT and mutated receptors were statistically significant; one-way ANOVA (*P* < 0.001); followed by multiple comparisons of the relative responses of mutated receptors vs. WT (*RI*_Spont_ 0.44 ± 0.06 (*n* = 24); *RI*_Glu_ 0.63 ± 0.30 (*n* = 24); *RI*_Gly_ 1.1 ± 0.2 (*n* = 24)); Dunnett’s test; *P < 0.050.

Figure 1C shows an example of activity recorded for WT, GluN1(ΔL655)/GluN2B, GluN1(ΔL657)/GluN2B receptors and GluN1(ΔP660)/GluN2B receptors. The responses of GluN1(ΔL657)/GluN2B were characterized by a high degree of spontaneous activity (*RI*_Spont_ = 75 ± 4%; *n* = 6) that was further increased by glycine (*RI*_Gly_ = 21 ± 4%; *n* = 6) or glutamate (*RI*_Glu_ = 10 ± 6%; *n* = 6). Deletions at the linker region distal to the M3 helix e.g., GluN1(ΔD658)/GluN2B exhibited less spontaneous activity, however both *RI*_Glu_ and *RI*_Gly_ were still present. Similarly, deletions at the M3-S2 linker of the GluN2B altered the channel gating. Figure 2A shows an example of the activity recorded for GluN1/GluN2B(ΔM654), GluN1/GluN2B(ΔI655) and GluN1/GluN2B(ΔY659) receptors. For GluN1/GluN2B(ΔI655) the *RI*_Spont_ (17 ± 1%;* n* = 4) was relatively small compared to *RI*_Gly_ (61 ± 4%; *n* = 4), and *RI*_Glu_ (32 ± 7%; *n* = 4). Similarly to GluN1, deletions at the linker region distal to the M3 helix e.g., GluN1/GluN2B(ΔY659) exhibited minimal spontaneous activity as well as *RI*_Glu_ and *RI*_Gly_.

Similarly to single deletions, the deletion of two adjacent AAs altered the receptor channel activity. In the case of GluN1(ΔL655; ΔV656)/GluN2B and GluN1(ΔL657; ΔD658)/GluN2B *RI*_Spont_ was high (85% and 83%, respectively) and *RI*_Gly_ and *RI*_Glu_ had only a minor effect (Supplementary Figure S1A). As the double deletions were introduced to the more distal part of the linker—e.g., GluN1(ΔE661; ΔE662)/GluN2B the spontaneous activity was diminished, however, *RI*_Gly_ and *RI*_Glu_ were still present 28 ± 0.2% (*n* = 3) and 19 ± 2% (*n* = 3), respectively. Similarly to GluN1, double deletions introduced to the GluN2B affected NMDAR channel gating, but in contrast to GluN1 *RI*_Spont_ was lower (1%–12%; Supplementary Figure S1B).

**Figure 3:**
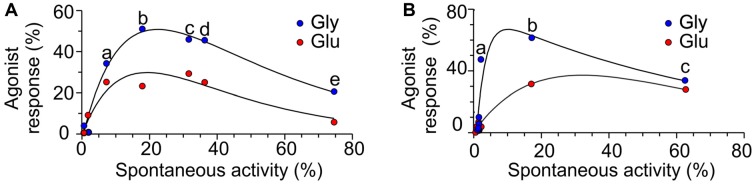
The relation between spontaneous activity and single agonist-induced responses.** (A)** Plot of the *RI*_Spont_ activity vs. *RI*_Glu_ (red symbols) and *RI*_Gly_ (blue symbols) in receptors with single-deletion mutations in GluN1 linker (*a* = ΔD658; *b* = ΔR659; *c* = ΔL655; *d* = ΔV656; *e* = ΔL657). **(B)** Plot of the *RI*_Spont_ activity vs. *RI*_Glu_ (red symbols) and *RI*_Gly_ (blue symbols) in receptors with single AA deletion mutations in GluN2B linker (*a* = ΔQ656; *b* = ΔI655; *c* = ΔM654).

Deletions in the M3-S2 linker of GluN1 and GluN2B subunits profoundly affect NMDAR channel function and the results summarized in Figures 1D, 2B allowed us to draw the following conclusions: (i) NMDARs with mutated linkers open spontaneously and as a consequence of receptor activation by a single agonist; (ii) the effect of deletions is stratified—spontaneous activity and single ligand-induced responses are more pronounced for deletions closer to the M3 helix; (iii) the degree of spontaneous activity and single-agonist responses, as well as the length of the linker region affected by deletions, differ for GluN1 and GluN2B subunits; (iv) irrespective of whether deletions have been introduced in GluN1 or GluN2B subunits, application of glutamate or glycine promoted receptor channel activity; (v) irrespective of whether deletions have been introduced to the M3-S2 linker of GluN1 or GluN2B, responses induced by glycine were (on average) larger than those induced by glutamate (Figures 3A,B); and (vi) none of the single and double deletions in the linker region mimicked maximal receptor activation (100%) by a single agonist (insensitive to glutamate and sensitive to glycine and vice versa insensitive to glycine and sensitive to glutamate).

To avoid activity induced by residual glycine (typical contamination of high-quality water and chemicals is in the concentration range of tens of nM, which is potentially not negligible considering that the glycine EC50 is nM for WT GluN1/GluN2B receptors, Chen et al., 2008), 7CKA was used as a competitive glycine site inhibitor (Kemp et al., 1988) in the above experiments. To exclude the possibility that 7CKA can on its own affect *RI*_Spont_ and *RI*_Glu_, we performed control experiments in which the activity was compared in solutions prepared in a way to maximally avoid glycine contamination and that containing 10 μM 7CKA. The values of *RI*_Spont_ recorded in the presence of 7CKA and in its absence were correlated (*r* = 0.997; *P* < 0.01) with a slope of 1.14 ± 0.02 (Supplementary Figure S2). Similarly, the values of *RI*_Glu_ recorded in the presence of 7CKA and in its absence were correlated (*r* = 0.604; *P* = 0.013) with a slope of 1.71 ± 0.30 (Supplementary Figure S2). These results indicated that 7CKA and/or background glycine only had a minor effect on gating of mutated NMDARs.

### Computational Analysis of Mutation-Induced Structural Changes in the M3-S2 Linker

To elucidate the molecular basis for the role of the M3-S2 linker in coupling the LBD to the ion channel, we employed computational methods. The presented model of the NMDAR is based on crystal structures of the heterotetrameric GluN1/GluN2B receptor (Karakas and Furukawa, 2014; Lee et al., 2014). Missing experimental electron density in the available crystal structures does not allow localization of residues in the linker regions and the related structural information is mostly missing; we, therefore, decided to explore linker structures computationally. The structural information for the linker regions, missing in the crystal structures, is a consequence of local disorder. This could be interpreted either by intrinsically disordered linkers not adopting any structure, or by a set of overlapping local structures and their transitions.

Conformational descriptors in terms of PB were used to reveal intrinsic structural preferences in the linker region (see Supplementary Figure S3A). PB are structural prototypes defined by de Brevern et al. (2000). Complex three-dimensional structural information of a protein backbone can be encoded as a one-dimensional sequence of PB “letters”. The PB cover all possible protein conformations and an automated procedure assigns each amino acid residue in a protein structure into one of the sixteen available PB. This procedure represents a significant extension of the commonly used secondary structure helix/sheet/coil descriptors, allowing fine analysis of protein flexibility (Craveur et al., 2015).

The results summarized as a PB structural alphabet logo indicate that only the first three residues in each linker (GluN1 LVL and GluN2B MIQ) have the potential to carry a high content of secondary structure (Supplementary Figure S3B). The consensus shows that these three residues cover the whole transition from helical to extended conformation, with the first residue visiting mostly helical or “end of helix” conformations, while the structure of remaining residues is already dominated by extended conformations. In general, the GluN1 linker structure is more disordered, while the structure of the GluN2B linker shows a higher content of extended conformations in the linker region. One potential problem with single AA deletions in the M3-S2 linker is that each may in principle induce multiple changes in the secondary structure that are difficult to predict. But the comparison of the M3-S2 linker between the WT and single AA deleted models revealed that single AA deletions in the GluN1(L655 to E662) as well as in the GluN2B(M654 to D661) had only a minor effect on the linker secondary structure. In theory, for a fully extended conformation, a single AA deletion induces shortening of the protein by ~3.4 Å; however, the extended conformation content is relatively low after averaging all the data, indicating that even the GluN2B linker is rather flexible and not stretched to its maximum length. Although the PB sequence logo is dominated by letters corresponding to extended conformations, the extended conformation is found only locally, not spanning over a longer patch of residues within the same model. There is also no evidence of shortening the M3-S2 distance by forming a patch of a more compact secondary structure element (like α-helix). Further, the effect of the deletion remains mostly local, compensated by conformation change of the nearest neighboring residues and does not propagate to the more distant regions.

### Effect of Substitutions in the M3-S2 Linkers of the GluN1 and GluN2B on Channel Gating

To further strengthen the hypothesis that altered NMDAR gating caused by AA deletions in the linker region is primarily due to the disruption of specific interactions at the linker-to-channel transition we decided to analyze the effect of glycine substitution of single AAs in the linker region. To rule out the effect of shortening we employed computational methods to analyze conformations of linkers with one AA substituted by glycine. Comparison of the M3-S2 linkers between WT and single-substituted models (Supplementary Figure S3C) revealed that single residue substituted by glycine in the GluN1(L655–E662) as well as in the GluN2B(M654–D661) had only a minor effect on the linker secondary structure and therefore it is unlikely that the linker length was considerably altered.

**Figure 4:**
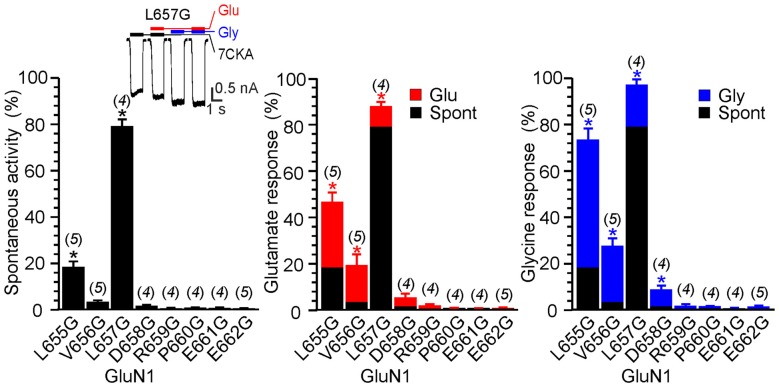
The effect of glycine mutagenesis of the GluN1 M3-S2 linker. Summary graphs of the mean ± SEM (*n*) of the relative responses—*RI*_Spont_ (black columns), *RI*_Glu_ (red columns) and *RI*_Gly_ (blue columns; see Equation 1–3)—in receptors with a single residue mutated to glycine in the GluN1 linker. Inset, representative current response of GluN1(L657G)/GluN2B. Application of ECS containing 7CKA (10 μM) with no added Mg^2+^, glutamate, or glycine is indicated by a black bar (7CKA); application of 1 mM glutamate and 7CKA (10 μM) with no added Mg^2+^ or glycine is indicated by a red bar (Glu); application of 0.1 mM glycine with no added Mg^2+^ or glutamate is indicated by a blue bar (Gly). In between the applications of Mg^2+^-free ECS the cells were bathed in ECS containing 2 mM Mg^2+^. Differences in the relative responses determined for WT and mutated receptors were statistically significant; one-way ANOVA (*P* < 0.001); followed by multiple comparisons of the relative responses of mutated receptors vs. WT (*RI*_Spont_ 0.44 ± 0.06 (*n* = 24); *RI*_Glu_ 0.63 ± 0.30 (*n* = 24); *RI*_Gly_ 1.1 ± 0.2 (*n* = 24)); Dunnett’s test; *P < 0.050.

**Figure 5:**
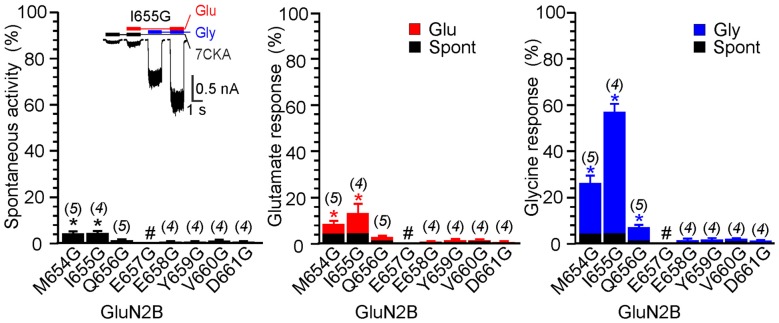
The effect of glycine mutagenesis of the GluN2B M3-S2 linker. Summary graphs of the mean ± SEM (*n*) of the relative responses–*RI*_Spont_ (black columns), *RI*_Glu_ (red columns) and *RI*_Gly_ (blue columns; see Equation 1–3)–in receptors with a single residue mutated to glycine in the GluN2B linker. Inset, representative current responses of GluN1/GluN2B(I655G). Application of ECS containing 7CKA (10 μM) with no added Mg^2+^, glutamate, or glycine is indicated by a black bar (7CKA); application of 1 mM glutamate and 7CKA (10 μM) with no added Mg^2+^ or glycine is indicated by a red bar (Glu); application of 0.1 mM glycine with no added Mg^2+^ or glutamate is indicated by a blue bar (Gly). In between the applications of Mg^2+^-free ECS the cells were bathed in ECS containing 2 mM Mg^2+^. Differences in the relative responses determined for WT and mutated receptors were statistically significant; one-way ANOVA (*P* < 0.001); followed by multiple comparisons of the relative responses of mutated receptors vs. WT (*RI*_Spont_ 0.44 ± 0.06 (*n* = 24); *RI*_Glu_ 0.63 ± 0.30 (*n* = 24); *RI*_Gly_ 1.1 ± 0.2 (*n* = 24)); Dunnett’s test; *P < 0.050. ^#^Indicates a nonresponding mutation.

Next, we prepared a series of single glycine substitution mutations in the GluN1(L655–E662) and GluN2B(M654–D661) linker regions and analyzed functional consequences for the NMDAR channel gating. Insets in Figures 4, 5 show an example of activity recorded in GluN1(L657G)/GluN2B and GluN1/GluN2B(I655G). While in GluN1(L657G)/GluN2B receptors *RI*_Spont_ was high and glutamate or glycine increased the current only a little, in GluN1/GluN2B(I655G) receptors *RI*_Spont_ was low and glutamate or glycine increased the current substantially. Other glycine substitution mutations in the linker region more distal to the M3 helix—GluN1(D658G to E662G) and GluN2B(E658G to D661G)–exhibited only minor spontaneous or single agonist-induced activity (Figures 4, 5). Functional consequences of glycine mutagenesis are similar to those of deletions, indicating on the importance of specific linker-linker interactions rather than simply altered linker length.

### Single-Channel Analysis of M3-S2 Linker Mutated Receptors

**Figure 6:**
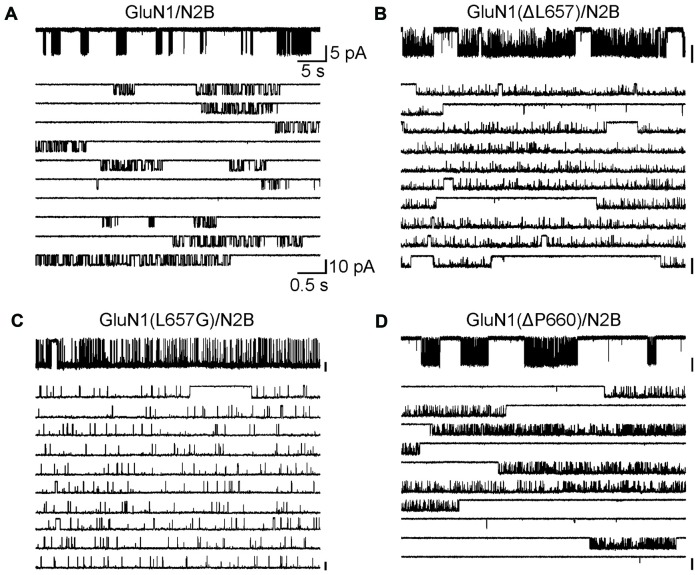
The GluN1(ΔL657) and GluN1(L657G) mutations alter single-channel properties. **(A–D)** Portions of steady-state cell-attached patch records, each containing only one active channel filtered at 1 kHz (for the purpose of the presentation only) from HEK293 cells expressing WT receptors **(A)**, GluN1(ΔL657)/GluN2B **(B)**, GluN1(L657G)/GluN2B **(C)**, or GluN1(ΔP660)/GluN2B **(D)** receptors. Each record is expanded below to 10 traces (1 kHz). Unitary currents were activated in these patches by 1 mM glutamate and 0.1 mM glycine.

The data in Figures 1–5 express the degree of impairment of mutated receptors in terms of a relative number reflecting receptor activation by glutamate and glycine co-application. It has been shown earlier that at saturating concentrations of glutamate and glycine GluN1/GluN2B WT receptors open with a low probability ~10% (Chen et al., 1999). Next, the single-channel analysis was used to assess the Po of mutated GluN1/GluN2B receptors as a measure of impairment of the NMDAR channel gating. For each receptor type, we obtained long-duration recordings (≥10^5^ events) of steady-state currents from cell-attached membrane patches containing only one active channel (Figure 6). Inward sodium currents were obtained in the presence of saturating concentrations of agonists (1 mM glutamate and 0.1 mM glycine) under conditions that minimized inhibitory effects of contaminating divalent ions (1 mM EDTA) at a holding potential +100 mV (estimated membrane potential ~ −130 mV). The records contained unitary currents of uniform amplitudes, with no obvious superimposed openings; typical currents occurred as bursts of frequent openings separated by long silent periods. First, we analyzed single-channel records to estimate Po, MOT and MCT, as global measures of gating kinetics for WT and mutated receptors (GluN1(ΔL657)/GluN2B and GluN1(ΔP660)/GluN2B). For comparison, we have also introduced a glycine mutation at the position GluN1(L657) to preserve the number of AAs within the linker, however with a minimal effect on the space occupied by L657 in the WT NMDAR. By these metrics, GluN1(L657G)/GluN2B and GluN1(ΔL657)/GluN2B receptors had, respectively, a 7.5-fold and a 5.7-fold higher Po relative to the WT receptor. For either mutated receptor, the higher activity was due to shorter closures (MCT 11- and 4.1-fold, respectively) and longer openings (MOT 12- and 4.3-fold, respectively; Figures 6A–C and Table 1). As expected, the receptors with deletions in the linker region distal to the M3 helix e.g., GluN1(ΔP660)/GluN2B exhibited Po values more comparable with WT receptor (Figure 6D and Table 1).

**Table 1:** Summary of single-channel properties.

	Po	MCT (ms)	MOT (ms)	Amplitude (pA)	Total events (*n*)
GluN1/GluN2B	0.12 ± 0.02	49 ± 11	5.1 ± 0.5	8.0 ± 0.4	4.4 × 10^5^ (6)
GluN1(ΔL657)/GluN2B	0.68 ± 0.09*	12 ± 4*	22 ± 5	6.3 ± 0.4	6.0 × 10^5^ (5)
GluN1(ΔP660)/GluN2B	0.21 ± 0.05	20 ± 3*	4.8 ± 0.6	7.2 ± 0.4	4.0 × 10^5^ (4)
GluN1(L657G)/GluN2B	0.90 ± 0.03*	4.6 ± 1.0*	63 ± 14*	7.2 ± 1.3	1.0 × 10^5^ (6)

The values represent the mean ± SEM (*n*) for each data set. *P < 0.05; one-way ANOVA, Dunnett’s test.

### LILI Motif and NMDAR Gating

Our results suggest that along the way to the ion channel opening, the M3-S2 linker transduces the signal from the LBD leading to the pore opening, however, not exclusively only by simple mechanical coupling. We hypothesize that the overall effect of the deletions and substitutions on the NMDAR gating we describe can be explained by an interaction of two pairs of specific M3-S2 linker residues at a site located in the proximity of the M3 helices (LILI motif) rather than a simple shortening of the linker. These interactions may form the basis for a novel extracellular NMDAR site controlling the process of gating. Mutations in either the GluN1 or the GluN2B M3-S2 linkers disrupt the receptor gating. Therefore, the NMDAR channel can open spontaneously and be activated to a similar extent by glutamate (in the absence of glycine) and by glycine (in the absence of glutamate).

**Figure 7:**
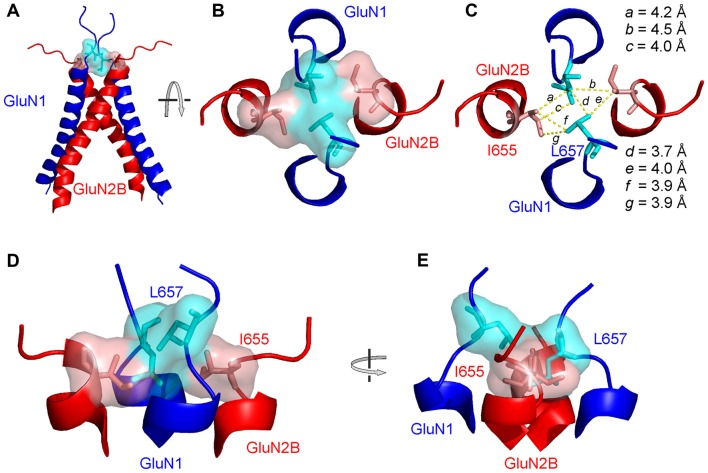
The structural arrangement of the NMDAR channel gate. **(A)** Ribbon diagram showing the GluN1 (blue) and GluN2B (red) M3 helices with the initial portion of the M3-S2 linkers. Semitransparent molecular surface representation of GluN1 L657 and GluN2B I655 is indicated in light blue and light red, respectively. **(B)** The extracellular channel gate of the GluN1/GluN2B receptor is shown in a closed conformation and viewed from the extracellular side of the membrane. **(C)** The same view is shown with molecular distances between L657 and I655 residues indicated.** (D,E)** The structure of the gate viewed from the side with the GluN1 M3 helices in the front and in the back **(D)** and rotated by 90° along the longitudinal axis **(E)**.

Measurements of spontaneous activity and single-agonist-induced responses in both the single-AA-deleted and glycine-substituted M3-S2 linker mutants agree on the importance of the GluN1 LVL as well as the GluN2B MIQ residues and indicate that the extracellular gating motif is located at this site close to the M3 helices. To understand the detailed structural organization of the NMDAR channel at its M3-to-linker transition we have further extracted and analyzed a set of lowest energy conformations (according to their DOPE score from MODELLER) for LVLDRPEE and MIQEEYVD linker residues from the above-mentioned NMDAR models. Within the LVL and MIQ regions, we have identified a specific role of the GluN1(L657) in the NMDAR channel gating. This explains the experimentally observed spontaneous activity after the deletion of this residue. On the other hand, the GluN2B(I655) serves as a potential binding partner to the gating GluN1(L657) (Figure 7). Further, comparing the structural propensities of the GluN1 and the GluN2B linkers close to the transmembrane helices, we can speculate that mutations in the already more flexible GluN1 induce an easier loss of helical structure and a higher solvent accessibility of the channel mouth and can lead to spontaneous activity.

Based on the residues involved in the extracellular gate we will use the “LILI” shortcut to address this motif throughout the text. The LILI motif in the gating conformation was identified within the most stable NMDAR models (Figure 7A, Supplementary Figure S4). An important structural feature of the LILI motif is that the two GluN1 leucine residues are not arranged symmetrically but rather stacked over each other, while both interacting with the GluN2B isoleucine residues (Figures 7B,D,E). This arrangement allows for tight van der Waals (vdW) interactions (alternatively called dispersion or hydrophobic) efficiently shielding the channel entrance from water (Figure 7C). It also offers an explanation for the opening mechanism involving the GluN1 linker with its unexpected orientation along the direction of the channel longitudinal axis. A naive model of the mechanism of opening a channel composed of four transmembrane helices would involve simultaneous pulling of all four linkers in the direction parallel to the membrane plane (which is the case only for the GluN2 linker).

**Figure 8:**
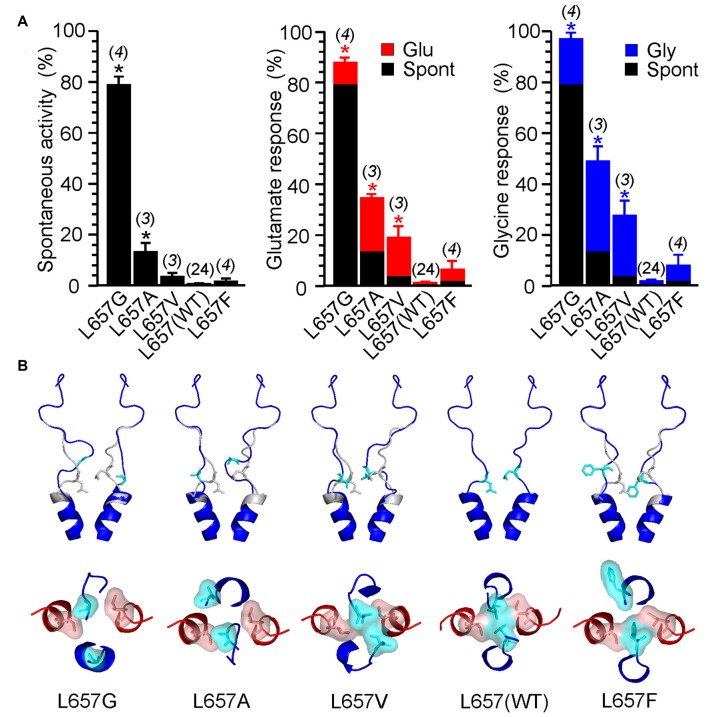
Consequences of GluN1(L657) mutations. **(A)** Summary graphs of the mean ± SEM (*n*) of the relative responses—*RI*_Spont_ (black columns), *RI*_Glu_ (red columns) and *RI*_Gly_ (blue columns; see Equations 1–3)—in receptors with a single residue GluN1(L657) mutated to glycine (G), alanine (A), valine (V), phenylalanine (F) and glutamine (Q). Differences in the relative responses determined for WT and mutated receptors were statistically significant; one-way ANOVA (*P* < 0.001); followed by multiple comparisons of the relative responses of mutated receptors vs. WT (*RI*_Spont_ 0.44 ± 0.06 (*n* = 24); *RI*_Glu_ 0.63 ± 0.30 (*n* = 24); *RI*_Gly_ 1.1 ± 0.2 (*n* = 24)); Dunnett’s test; *P < 0.050. **(B)** Ribbon representation of GluN1 subunits (blue) and GluN2B subunits (red) at the extracellular channel vestibule viewed from the membrane and the extracellular side. The computational models show the most frequent (typical) position (structure) of GluN1(L657) in the WT (indicated in gray) and when mutated to glycine (G), alanine (A), valine (V), leucine—wild type (L) and phenylalanine (F; indicated in blue).

The stability of the LILI motif can be attributed to a tight vdW interaction of the nonpolar leucine and isoleucine residues. To further strengthen this hypothesis, we used the NMDAR model to test the structural to functional consequences of the GluN1(L657) substitution for glycine, alanine, valine, phenylalanine and glutamine (Figure 8 and Supplementary Figure S5). The selection includes nonpolar amino acids with increasing size and hydrophobicity of the side chain as well as a polar glutamine of a size similar to the substituted leucine. The results of the simulation indicate that when GluN1(L657) was substituted by a small AA glycine, the vdW interaction that existed in the LILI motif was substantially weakened and the introduced GIGI motif became unstable with the expected loss of gating ability (Figure 8B, L657G). This is in agreement with the experimentally observed high degree of spontaneous activity (Figures 4, 8A, Supplementary Figure S5A, L657G). The substitution of GluN1(L657) for alanine and valine allowed partial restoration of the vdW interaction between the residues. However, the interaction with the GluN2B(I655) led to the partial unfolding of the GluN1 M3 helix (Figure 8B, L657A, L657V). The modeling of the L657F substitution revealed a favorable interaction of the larger phenylalanine residue with GluN2B(I655), although the other phenylalanine residue was not in direct interaction due to steric constraints (Figure 8B, L657F). Mutations in this series of hydrophobic amino acids markedly reduced spontaneous activity when compared to that observed for the GluN1(L657) substitution by glycine (Figure 8A and Supplementary Figure S5A). The activity induced by glutamate or by glycine decreased in a similar order as spontaneous activity (L657A > L657V > L657F, Figure 8A, Supplementary Figure S5A) and this agrees well with the computational prediction. In the case of the GluN1(L657) substitution for glutamine, vdW interaction is expected to be negligible and as a consequence, the polar glutamine was expelled and oriented towards the water environment (Supplementary Figure S5B, L657Q). The functional examination showed an increase of spontaneous and single-agonist-evoked activity of the glutamine mutated NMDAR (Supplementary Figure S5A, L657Q).

In addition, the role of the LILI motif in NMDAR gating is supported by experiments in which leucine and isoleucine were substituted by cysteine. Reducing agent dithiothreitol (DTT) potentiated WT receptors by 93 ± 7% (*n* = 6) while GluN1(L657C)/GluN2B(I655C) receptor responses were potentiated significantly more, by 739 ± 84% (*n* = 4; *t*-test *P* < 0.001). The effect of DTT was reversible upon application of oxidizing agent 5,5′-dithiobis(2-nitrobenzoic acid) (DTNB; see Supplementary Figure S6).

## Discussion

Our results indicate that an extracellular LILI motif which is formed by the M3-S2 linkers (at the transition between the M3 helices and the linkers) is an important element that connects conformational changes at the level of the LBD induced by agonist binding and unbinding to the NMDAR channel opening and closing. We propose that the LBD pulling energy displaces GluN1 and GluN2 M3-S2 linkers which leads to disassembly of the compact form of the LILI motif which then serves as the first step in conformational transitions leading to the M3 helix separation, displacement, and finally to channel opening. In addition to classic NMDAR ion channel action, unconventional NMDAR signaling has been described recently including metabotropic NMDAR signaling (Nabavi et al., 2013; Dore et al., 2016, 2017; Bouvier et al., 2018). Since some form of NMDAR dependent long-term depression can be induced without Ca^2+^ influx, we suppose that altered ion channel gating would have a minor effect on the metabotropic action of NMDAR (to confirm this conclusion is beyond the scope of this work).

NMDAR channel opening occurs as a consequence of glycine binding to the GluN1 subunit and glutamate binding to the GluN2 subunit (Kuryatov et al., 1994; Laube et al., 1997). The results of our experiments in which the linker length was shortened or structure altered indicate that the GluN1 and the GluN2B M3-S2 linkers contribute unequally to channel opening and are consistent with previously published data in which the linker length was prolonged (Kazi et al., 2014) and when residues were substituted by alanine (Chang and Kuo, 2008). However, the exact relationship between the degree of individual linker displacement and the degree of channel opening remains to be resolved. Indeed, our data indicate that the deletion of a single AA at the linker site outside the gate already has a profound effect on channel gating (Figures 1, 2). The exact prediction of the possible linker shortening induced by a single AA deletion strongly depends on local linker structure, however, the upper limit for a fully extended structure of one AA is about 3.4 Å and a common shortening obtained from the analysis of our models is on the order of 1.5 Å. Our recently published data indicate that the extracellular NMDAR channel vestibule has to open widely (the diagonal C_α_ to C_α_ distance of 37 × 38 Å at the level of GluN1(D658) and GluN2B(E657)) to open the ion channel at the site of the TTTT ring (GluN1 T648 and GluN2B T647) to the minimum cross-section of the channel permeation pathway in the conducting states (Vyklicky et al., 1988, 2015). A similarly widely opened channel vestibule was proposed for the NMDAR just prior to channel opening (Dai and Zhou, 2013). This makes it likely that the degree of linker displacement and the degree of the channel opening at the vestibule are not directly linked. This is further supported by crystallographic data indicating that the movement evoked at the level of the S2 lobe by agonist binding (Furukawa et al., 2005; Karakas and Furukawa, 2014; Lee et al., 2014), which is expected to be responsible for the linker pull, is relatively small.

The LILI motif represents a common hub for the mechanical pulling by the GluN1 and the GluN2 M3-S2 linkers. The nature of these forces is precisely balanced such that only joint mechanical pulling from both pairs of linkers allows the compact form of the LILI motif to disassemble and subsequently to initiate channel opening. On the structural level, the gate must be fine-tuned and even small changes (Figure 8B) have a profound effect on channel gating. Previous crystallographic and functional studies suggested an additional site located within the highly conserved SYTANLAAF motif of the M3 helix of both the GluN1 and the GluN2 subunits (Kashiwagi et al., 2002; Sobolevsky et al., 2007, 2009; Chang and Kuo, 2008; Vyklicky et al., 2015). This region contains the narrowest portion of the ion channel—the TTTT ring, the group of four threonines GluN1(T648) and GluN2B(T647) (Beck et al., 1999; Sobolevsky et al., 2002). Surprisingly, NMDARs with threonines mutated to alanines (GluN1(T648A) or GluN2B(T647A)) formed constitutively (spontaneously) open channels (Kashiwagi et al., 2002; Sobolevsky et al., 2007; Chang and Kuo, 2008; Vyklicky et al., 2015). This indirectly indicates the role of the TTTT ring in the stabilization of the closed conformation of the NMDAR channel. This is also in line with computational approaches indicating a compact geometry at the level of the TTTT ring that allows optimal distance to form vdW contacts as well as hydrogen bonds among the side chain atoms of the threonine residues (Vyklicky et al., 2015).

**Figure 9:**
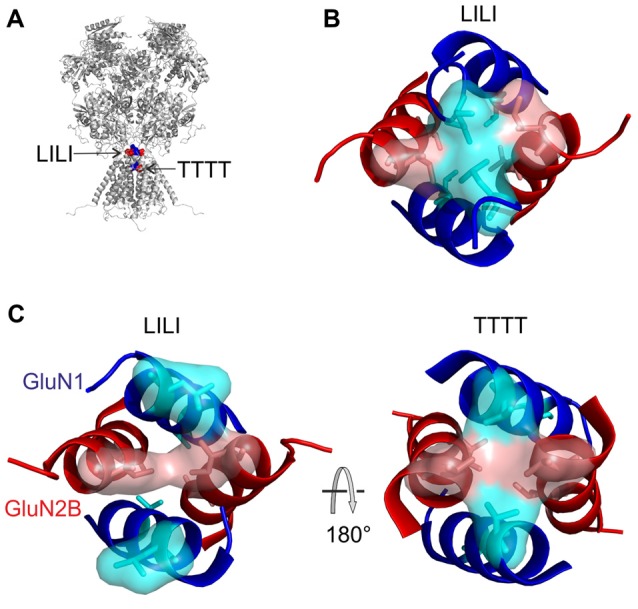
Model of the NMDAR channel opening. **(A)** The location of LILI and TTTT gates is shown. **(B)** The LILI gate in the activated closed state serves as a “plug” shielding the channel entrance from the water. During the opening, the upward pulling of the GluN1 M3-S2 linker resulting from glycine binding and the sideward pulling of the GluN2 M3-S2 linker resulting from glutamate binding, as induced by the ligand-binding domain (LBD) conformational change, leads to the breaking of the tight hydrophobic LILI-gate, exposing the channel entrance to water. **(C)** An intermediate state involving the formation of the second gate, the TTTT ring. However, once water reaches the TTTT ring, it competes with the hydrogen bonding and the vdW contacts among the threonines and as a consequence the M3 helices separate, leading to the open NMDAR channel.

We have employed computational methods (molecular modeling features of MODELLER package, including the conjugate gradient optimization and molecular dynamics) to reveal the individual molecular steps in the process of NMDAR channel opening. The available crystal structures of the heterotetrameric GluN1/GluN2B receptor were solved with agonists bound (Furukawa et al., 2005; Karakas and Furukawa, 2014; Lee et al., 2014) and therefore it is likely that they represent a transitory state of the channel just prior to its opening—an activated closed state. Although we cannot completely exclude the speculation that the crystal structure could also represent a desensitized state, our observation of the asymmetric behavior of NMDAR models at the channel entrance might suggest that the crystal structure represents an average of the oscillating movements of the M3 helices. At the level of the LILI motif, this state is characterized by vdW interactions between diagonally located leucines (L657) of the GluN1 subunits and adjacent isoleucines (I655) of the GluN2B subunits (Figures 9A,B). The opening of the channel was modeled similarly to our previous work (Vyklicky et al., 2015) by stepwise elongation of the distance between pairs of GluN1 and GluN2B domains anchored by the Cα atoms of the topmost residues within the pore-forming helices. As discussed previously, the TTTT ring (Figure 9A) does not form a tight interaction in the available crystal structures and this interaction is formed only during the process of NMDAR opening. A more detailed picture of the NMDAR opening process involves the currently proposed LILI motif. For the modeling, the initial, crystal-derived geometry of the transmembrane helices was identical to the previous work and the currently used model differed only in the newly identified geometry of the linkers. The modeling connected the starting LILI model with the previously described TTTT ring intermediate and the fully opened NMDAR channel.

Based on the modeling we predict that the upward pulling of the GluN1 M3-S2 linker resulting from glycine binding and the sideward pulling of the GluN2 M3-S2 linker (see Figure 1B for linker orientation) resulting from glutamate binding leads to the breaking of the tight hydrophobic interaction in the LILI motif that serves as a “plug” thus exposing the channel entrance to water (Figure 9C, *left*). The breaking of the interaction in the LILI motif results from a combination of the separation of the outer segments of the M3 helices and the rotation of these helices induced by pulling the M3-S2 linkers. The modeling suggests that during the reorganization at the LILI motif level the originally more separate TTTT residues form a tightly interacting ring (Vyklicky et al., 2015). However, once water reaches the TTTT ring (Figure 9C, *right*) it competes with the hydrogen bonding and the vdW contacts among the threonines and as a consequence, the M3 helices separate and form a bistable state oscillating between open and closed conformations lasting for the duration of the receptor activation by agonists. In this model, the LILI gate and TTTT ring form a functionally interconnected unit that controls the NMDAR channel gating.

From a different point of view, the GluN1(L657) is located in the vulnerable region of the receptor where structural changes (Figure 8) have profound functional consequences. Recent human genetic studies show the existence of multiple alterations in genes encoding NMDAR subunits (GRIN1, GRIN2A-B; Yuan et al., 2015; Hu et al., 2016). Missense mutations localized in the M3-S2 linkers of GluN1, GluN2A-C, and GluN3A-B subunits (GluN1(L655–D669) three missense mutations; GluN2A(M653-D667) one missense mutation; GluN2B(M654–D668) three missense mutations; GluN2C(M651–D665) four missense mutations; GluN3A(M768–D782) four missense mutations; GluN3B(M668–D682) three missense mutations; *note—no mutations were observed in GluN2D(M678–D692)*) were found (ClinVar^1^, accessed 29-3-2018; Exome Aggregation Consortium, Cambridge, MA^2^, accessed 29-3-2018; and UCSC Genome Browser^3^, accessed 29-3-2018). These mutations are associated with clinical symptomatology such as mental retardation (GluN1(E662K); Hamdan et al., 2011); motor delay, delayed speech and language development (GluN2B(D668N)), and/or are likely pathogenic (GluN2B(E657G, D668Y); ClinVar^1^). Since the effects of LBD and TMD alterations are bidirectional, missense mutations may affect not only channel gating but also LBD cleft closure and thus change agonist potency, deactivation time course, and sensitivity to competitive antagonists and allosteric modulators (Jones et al., 2002; Sobolevsky et al., 2002; Yuan et al., 2005). This, together with the data presented here, indicates the structure-functional importance of the linker region and implies that receptor malfunction manifests by divergent clinical symptomatology. Finally, our data also provide a structural basis for actions of compounds affecting the efficacy of signal transduction from the LBD to the NMDAR channel gating and thus may promote the development of new drugs for treatment NMDAR channelopathies associated with severe neurological and psychiatric symptoms.

Here we identify a novel step in the gating mechanism of the NMDAR. Multidisciplinary approach was used to show that amino acids localized at the transition of the M3-S2 linkers to the M3 helices of the GluN1 and GluN2B are critically involved in stabilizing the ion channel in the closed conformation and further that GluN1(L657) and GluN2B(I655) complement the function of the previously identified GluN1(T648) and GluN2B(T647) gating residues located in the ion channel. These results provide insight into the structural organization of M3-S2 linkers and the functional interplay of the LILI motif with the TTTT ring during the course of NMDAR channel opening and closing.

## Author Contributions

AB and LV designed the study. ML and JK carried out and analyzed the electrophysiology experiments. JC performed molecular modeling. AB, MH and LV wrote the article.

## Conflict of Interest Statement

The authors declare that the research was conducted in the absence of any commercial or financial relationships that could be construed as a potential conflict of interest.

## Footnotes

^1^https://www.ncbi.nlm.nih.gov/clinvar

^2^http://exac.broadinstitute.org

^3^https://genome.ucsc.edu/

## Abbreviations

7CKA: 7-Chlorokynurenic acid
AA: amino acid
DTNB: 5,5′-dithiobis(2-nitrobenzoic acid)
DTT: dithiothreitol
ECS: extracellular solution
HEK: human embryonic kidney
iGluRs: glutamate-activated ionotropic receptors
LBD: ligand-binding domain
MCT: mean closed time
MOT: mean open time
NMDAR: N-methyl-D-aspartate receptor
Po: channel open probability
RI_Glu_: relative glutamate current
RI_Gly_: relative glycine current
RI_Spont_: relative spontaneous current
TMD: transmembrane domain
WT: wild-type.
