# Supplementary material for: The LILI Motif of M3-S2 Linkers Is a Component of the NMDA Receptor Channel Gate
Source: Front Mol Neurosci. 2018 Apr 6;11:113. doi: 10.3389/fnmol.2018.00113 (PMC5897735; doi:10.3389/fnmol.2018.00113)
Supplement: Supplementary file 1 [file Data_Sheet_1.docx]

## Supplementary Material

**The LILI motif of M3-S2 linkers is a component of the NMDA receptor channel gate**

**Author listing:**

Marek Ladislav^1,2,3^, Jiri Cerny^1,3^, Jan Krusek^1^, Martin Horak^1^, Ales Balik^1^, and Ladislav Vyklicky^1^

^1^ Institute of Physiology CAS, Videnska 1083, 142 20 Prague 4, Czech Republic

^2^ Department of Physiology, Faculty of Science, Charles University in Prague, Albertov 6, Czech Republic

^3^ Co-first authors


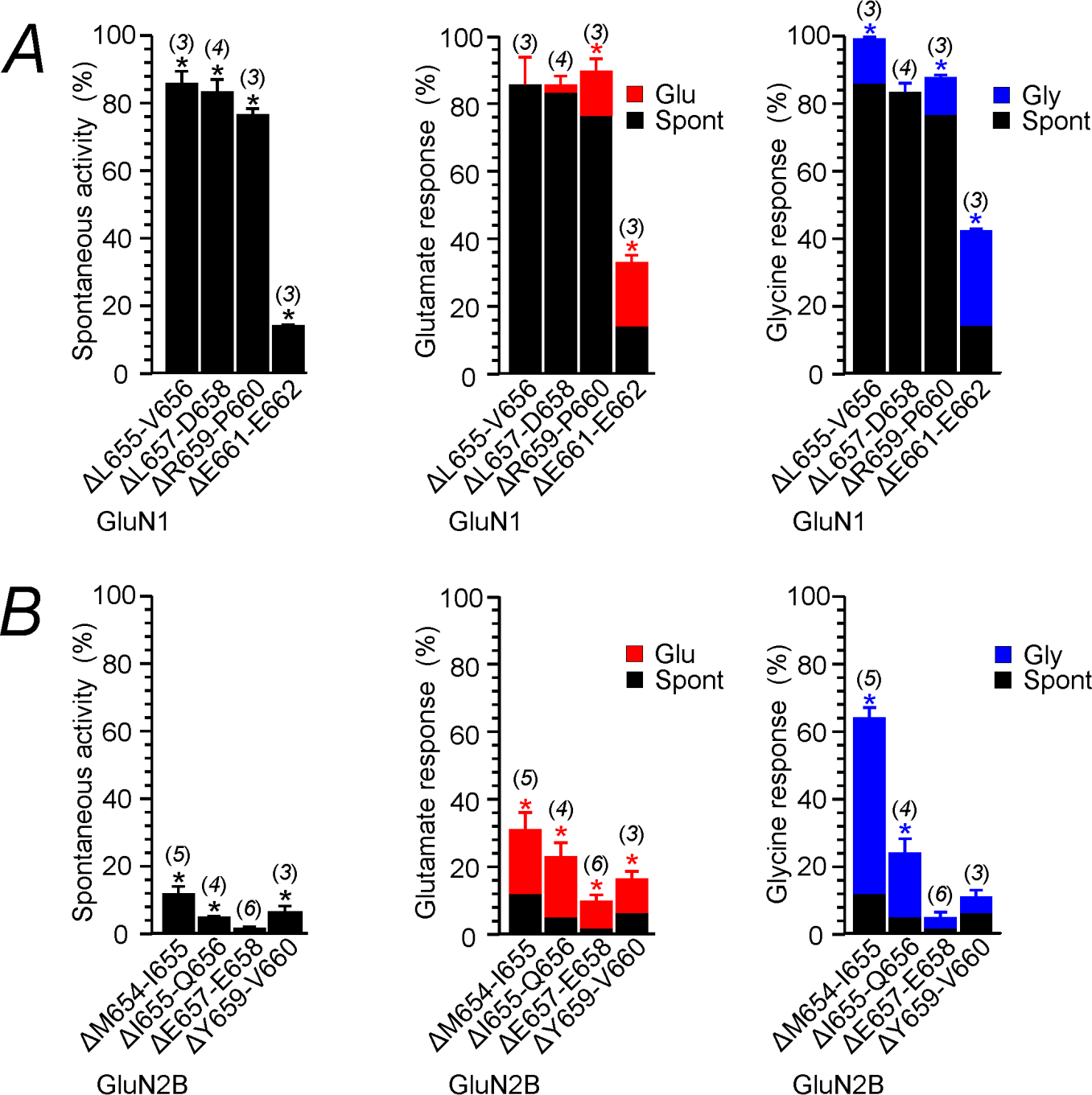


#### Supplementary Figure S1

The effect of double-deletion mutations of the M3-S2 linkers. (**A** and **B**) The effect of double-deletion mutations of the M3-S2 linker of GluN1 or GluN2B on receptor activity. Summary graphs of the mean ± SEM (*n*) of the relative responses - ${RI}_{Spont}$ (black columns),${RI}_{Glu}$(red columns), and ${RI}_{Gly}$ (blue columns) (see Equations 1-3) - in receptors with double deletion mutations at the M3-S2 linker of GluN1 (**A**) or GluN2B (**B**). Differences in the relative responses determined for WT and mutated receptors were statistically significant; one-way ANOVA (P <0.001); followed by multiple comparisons of the relative responses of mutated receptors *versus* WT (${RI}_{Spont}$ 0.44 ± 0.06 (*n* = 24); ${RI}_{Glu}$0.63 ± 0.30 (*n* = 24); ${RI}_{Gly}$ 1.1 ± 0.2 (*n* = 24)); Dunnett's test; * P <0.050.


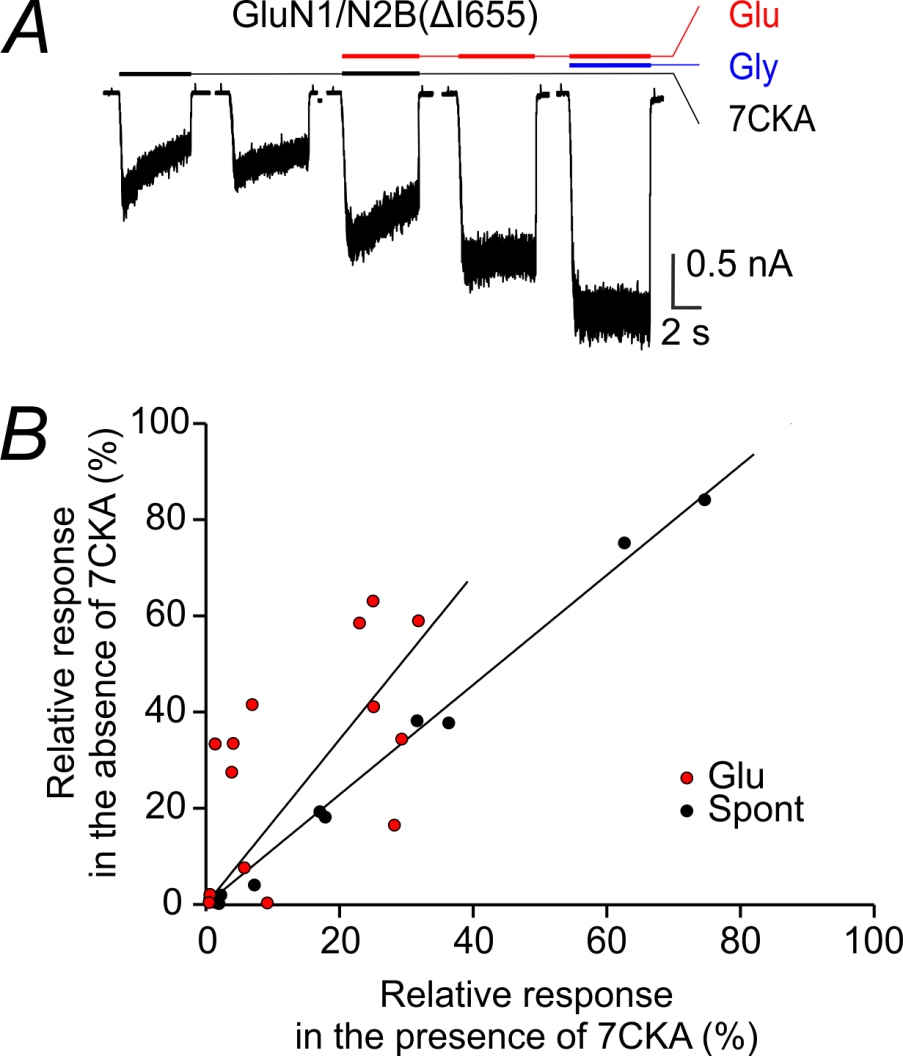


#### Supplementary Figure S2

Effect of 7CKA on NMDAR gating. (**A**) Representative current responses of GluN1/GluN2B(ΔI655). (**B**) ${RI}_{Spont}$ (•) and ${RI}_{Glu}$ (•) were recorded in the presence of 10 µM 7CKA (abscissa) and in the absence of 7CKA (ordinate) from NMDARs with single deletions introduced in the M3-S2 linker. ${RI}_{Spont}$recorded in the presence and absence of 7CKA were correlated (r = 0.998; p < 0.01), similarly to ${RI}_{Glu}$ recorded in the presence and absence of 7CKA (r = 0.604; p = 0.0133). Data from receptors carrying mutated GluN1 subunit (ΔL655-ΔE662; and GluN2B subunit (ΔM654-ΔD661) were pooled.


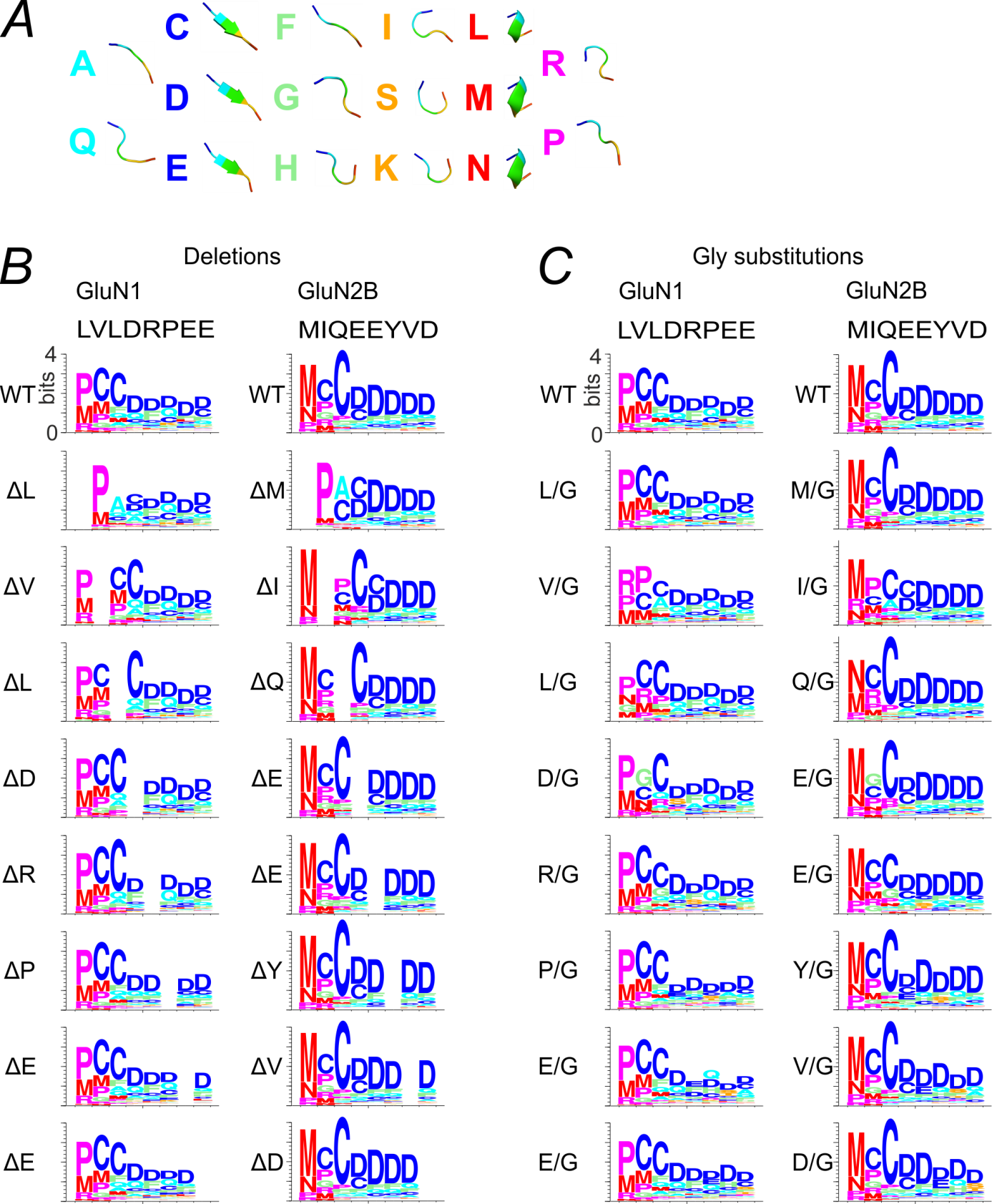


#### Supplementary Figure S3

Consequences of deletions or substitutions for the M3-S2 linker structure. (**A**) PB alphabet. Colored code corresponds to helical structures in red and extended structures in blue. (**B**) PB structural alphabet logos for single-residue deletions in the LVLDRPEE motif of the M3-S2 linker of GluN1 and the MIQEEYVD motif of GluN2B. (**C**) Logos for single-residue substitutions by glycine in the LVLDRPEE motif of the M3-S2 linker of GluN1 and the MIQEEYVD motif of GluN2B***.***

***
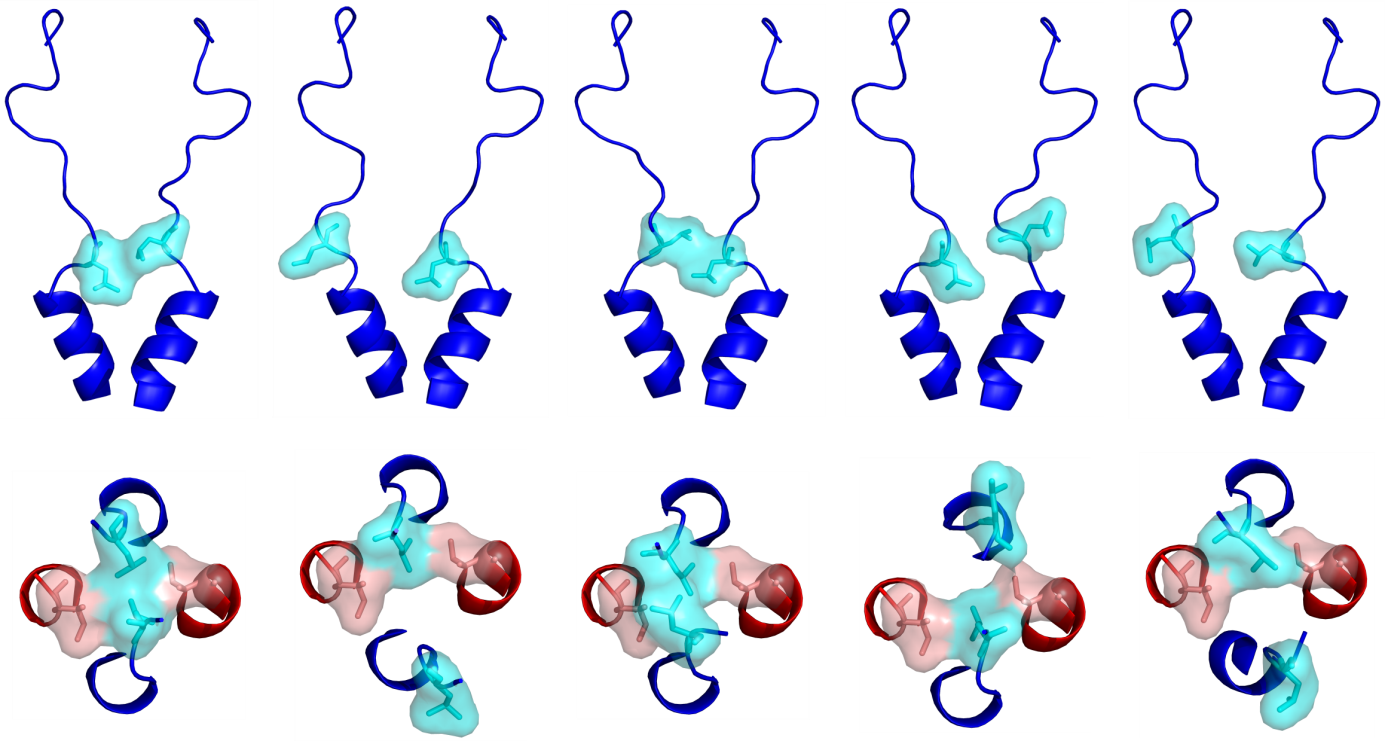
***

#### Supplementary Figure S4

The five most stable NMDAR models of LILI motif. Ribbon representations of GluN1 subunits (blue) and GluN2B subunits (red) at the extracellular channel vestibule viewed from the membrane and the extracellular side. The computational models show the most frequent (typical) position (structure) of GluN1(L657) and GluN2B(I655) in the NMDAR. Note that the two GluN1 leucine residues are not arranged symmetrically but rather stacked over each other.

**
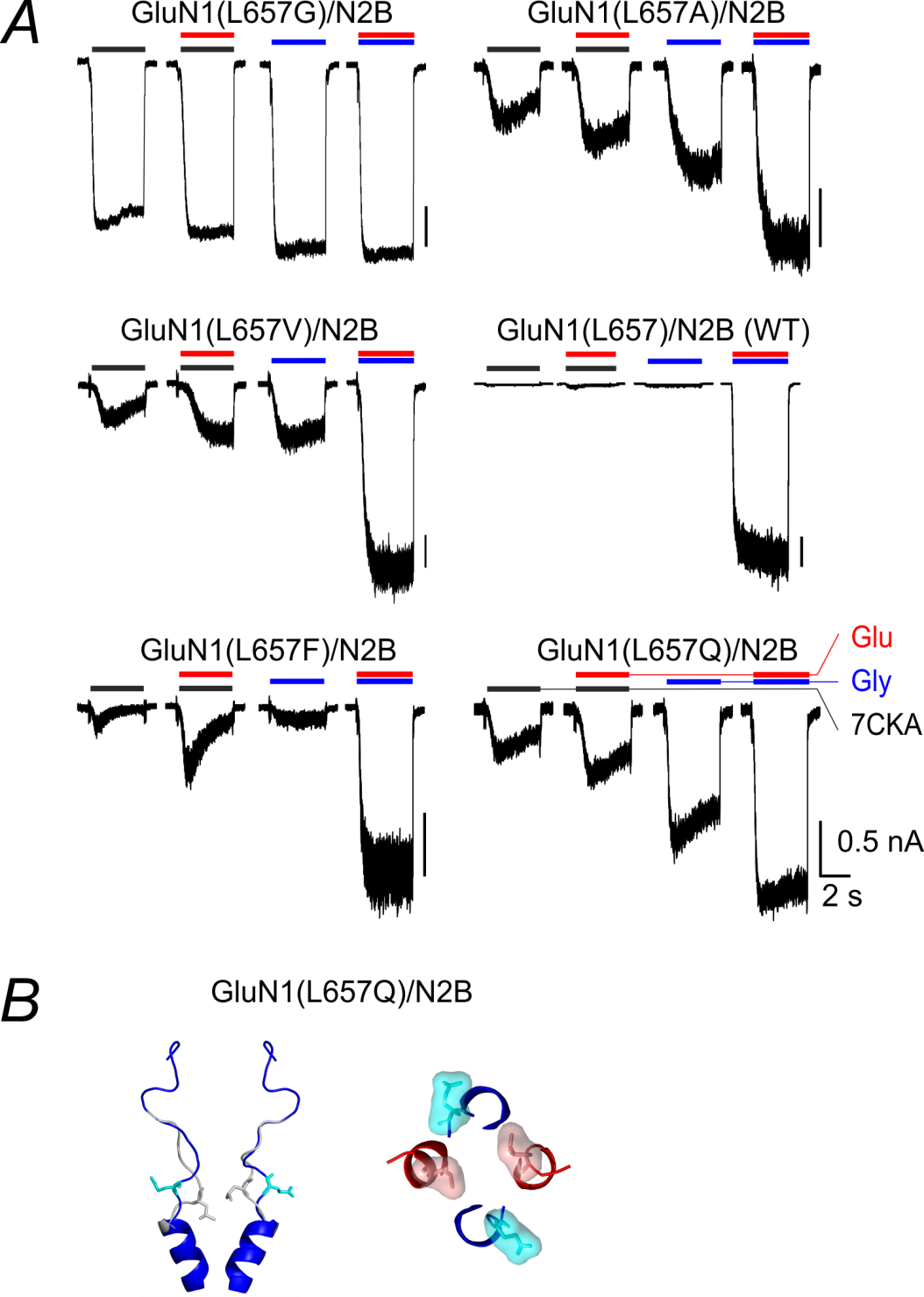
**

#### Supplementary Figure S5

Structural and functional consequences of GluN1(L657) mutations. (**A**) Representative current responses of GluN1(L657G)/GluN2B, GluN1(L657A)/GluN2B, GluN1(L657V)/GluN2B, WT GluN1/GluN2B, GluN1(L657F)/GluN2B and GluN1(L657Q)/GluN2B. Application of ECS containing 7CKA (10 µM) with no added Mg^2+^, glutamate, or glycine is indicated by a black bar (7CKA); application of 1 mM glutamate and 7CKA (10 µM) with no added Mg^2+^ or glycine is indicated by a red bar (Glu); application of 0.1 mM glycine with no added Mg^2+^ or glutamate is indicated by a blue bar (Gly). In between applications of the Mg^2+^-free ECS, the cells were bathed in ECS containing 2 mM Mg^2+^. (**B**) Ribbon representation of GluN1(L657Q)/GluN2B with GluN1 subunits (blue) and GluN2B subunits (red) at the extracellular channel vestibule viewed from the membrane and the extracellular side. The computational model shows the most frequent (typical) position (structure).


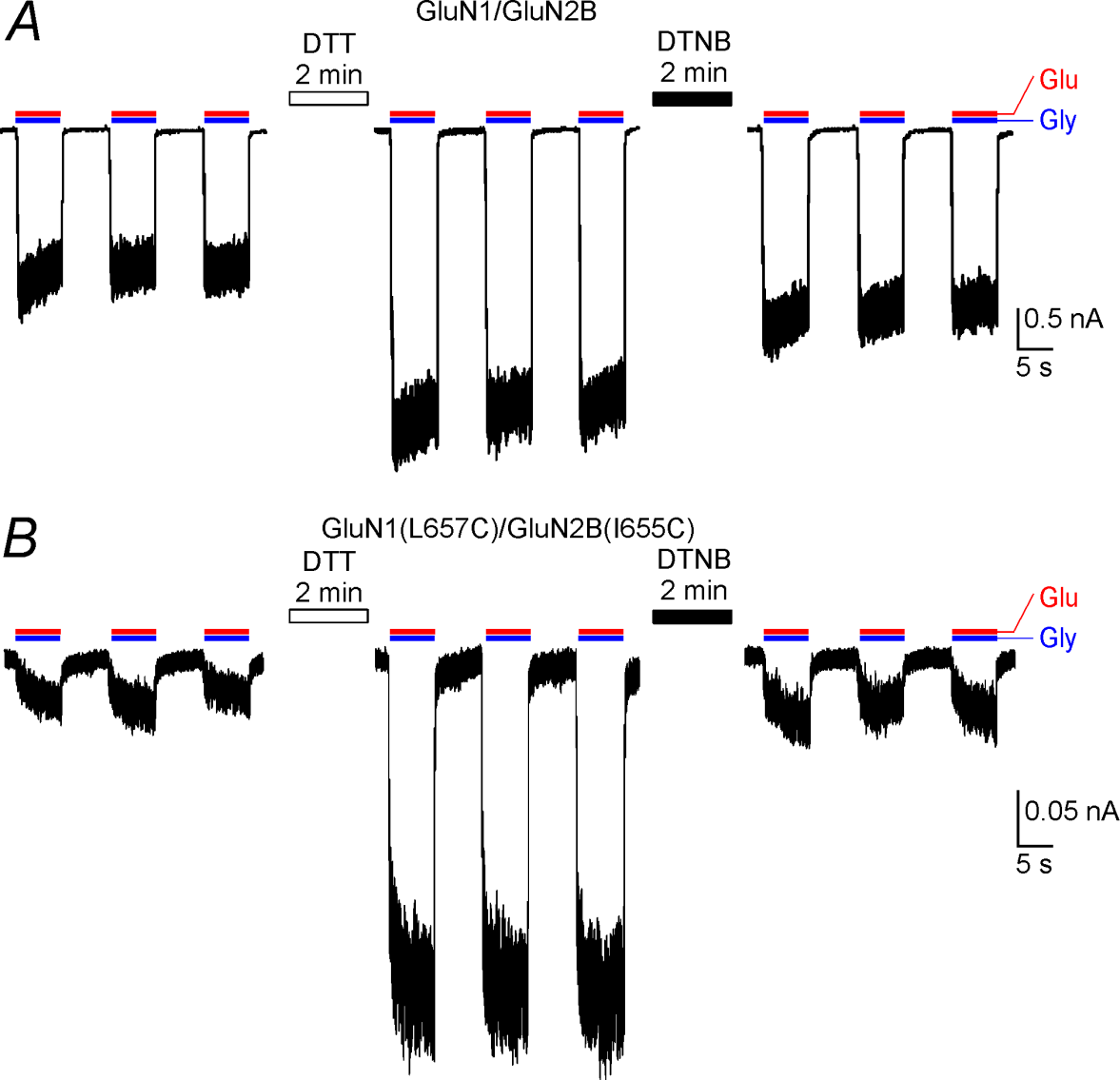


#### Supplementary Figure S6

The functional effect of cysteine mutations in the LILI motif. The representative current responses of WT **(A)** and GluN1(L657C)/GluN2B(I655C) receptors **(B)**. Three responses were induced by 1 mM glutamate (red bar) and 0.1 mM glycine (blue bar) recorded in the absence of Mg^2+^. The open bar indicates the application of 4 mM reducing reagent dithiothreitol (DTT) for 2 min and the filled bar indicates the application of 1 mM oxidizing agent 5,5′-dithiobis(2-nitrobenzoic acid) (DTNB) for 2 min.
